# The association between physical activity with incident obesity, coronary heart disease, diabetes and hypertension in adults: a systematic review of longitudinal studies published after 2012

**DOI:** 10.1186/s12889-020-08715-4

**Published:** 2020-05-19

**Authors:** Laura Cleven, Janina Krell-Roesch, Claudio R. Nigg, Alexander Woll

**Affiliations:** 1 Institute of Sports and Sports Science, Karlsruhe Institute of Technology, Karlsruhe, Germany; 2 Translational Neuroscience and Aging Laboratory, Mayo Clinic, Scottsdale, AZ USA; 3 Present address: Institute of Sports Science, University of Bern, Bern, Switzerland

**Keywords:** Physical activity, Obesity, Coronary heart disease, Diabetes, Hypertension, Adults, Longitudinal study, Cohort study

## Abstract

**Background:**

A growing body of studies that investigated the longitudinal association between physical activity (PA) and the outcome of incident obesity, coronary heart disease (CHD), diabetes and hypertension has become available in recent years. Thus, the purpose of this systematic review was to provide an update on the association between PA and onset of obesity, CHD, diabetes and hypertension in individuals aged ≥18 years who were free of the respective conditions at baseline.

**Methods:**

We systematically searched OVID, Pubmed, and Web of Science databases for pertinent literature published between January of 2012 and February of 2019. To ensure that conclusions are based on high quality evidence, we only included longitudinal studies conducted in samples of ≥500 participants and with ≥5 years of follow-up.

**Result:**

The search yielded 8929 records of which 26 were included in this review. Three studies were conducted on the outcome of incident obesity, eight on incident CHD, nine on incident diabetes, four on incident hypertension, one on the outcome of both diabetes and hypertension, and one on the outcome of CHD, diabetes and hypertension. Overall, there was an association between PA and lower risk of incident obesity, CHD and diabetes, but not hypertension. Higher levels or amount of PA were associated with a reduced risk of new onset of the respective diseases in 20 studies (77%). Whereas four studies reported an elevated risk of incidence of diseases with lower PA levels (15%). PA was not associated with incidence of diseases in two studies (8%).

**Conclusion:**

Higher levels of PA are likely associated with a lower risk of becoming obese, develop CHD or diabetes. These findings replicate and strengthen conclusions from earlier reviews underlining the importance of promoting PA in adults. The associations between PA and incident hypertension were less consistent. More research, particularly using prospective cohort designs in large population-based samples, is needed to further untangle the association between PA and incident hypertension.

**Trail registration:**

CRD42019124474 (PROSPERO Protocol registration). Date of registration in PROSPERO 27 February 2019.

## Background

The World Health Organization identified non-communicable diseases (NCDs), such as diabetes mellitus or cardiovascular diseases to be a major threat to economies and societies [1]. NCDs are implicated in 73% of all global deaths in 2017, with 28.8 million deaths attributed to risk factors like high blood pressure, high blood glucose, or high body mass index (BMI) [2]. Furthermore, NCDs are forecasted to account for 81% of all global deaths in 2040 [3].

NCDs usually develop over a long time period and may be impacted by an individual’s health behaviors [4]. As such, many NCDs may be preventable by decreasing metabolic risk factors such as hypertension, overweight and obesity, or hyperglycemia, as well as by decreasing behavioral risk factors like tobacco or alcohol use, an unhealthy diet, and physical inactivity [1, 5].

A growing body of research suggests that high levels of physical activity (PA) may have a protective effect on various health conditions including but not limited to overweight and obesity [6], coronary heart disease (CHD) [7, 8], type 2 diabetes mellitus [9, 10], hypertension [11, 12], and hyperglycemia [13, 14]. In addition, several longitudinal studies have become available that examine the association between PA and new onset of NCDs [15–20].

The current systematic review presents an update of a previously published review by our group [20], that examined the long-term effects of PA on type 2 diabetes mellitus, CHD, overweight/obesity and dementia by including studies published before 2012. Given the high significance of this topic and since we expected a substantial amount of relevant studies published after 2012, we provide an updated review of longitudinal studies on the association between PA and incident obesity, CHD, diabetes and hypertension over the past 7 years. Hypertension has been added to the current review as it is widely regarded as a major risk factor for several NCDs [21].

## Methods

This review was conducted in accordance with the Preferred Reporting Items for Systematic Reviews and Meta-Analysis (PRISMA) guideline [22]. The protocol was registered in the PROSPERO register of systematic reviews (CRD42019124474).

### Search strategy

Pertinent articles published between January of 2012 and February of 2019 were searched in electronic databases (PubMed, Web of Science and EMBASE by OVID) by applying a combination of one or more of the following search terms: “longitudinal and/or long-term”; “physical activity/exercise”; “adult”; “overweight and/or obesity”, “coronary heart disease and/or coronary artery disease and/or ischemic disease”, “diabetes mellitus and/or diabetes type 2”, “hypertension and/or blood pressure”. Both titles and abstracts were searched. After identification of studies, their bibliographies were searched manually to identify additional relevant studies.

### Study inclusion & exclusion criteria

We defined the following inclusion criteria for this systematic review: (1) Longitudinal, i.e. prospective cohort study design; (2) Studies reporting the association between PA and new onset of obesity, CHD, type 2 diabetes mellitus and/or hypertension; (3) Studies providing information on the assessment of PA (predictor variable). PA could be leisure-time/habitual PA, work-related PA, transportation related PA, organized and unorganized PA, etc.; (4) Only studies with ≥5 years of follow-up were included to allow for a meaningful conclusion on the longitudinal association between PA and selected outcomes of interest; (5) Studies with males and females aged ≥18 years, that were free of the diseases of interest at baseline; (6) Studies with more than 500 participants were included, to improve the probability to capture a substantial amount of incidence cases; and (7) articles written in English.

Excluded from this review were (1) studies investigating the effect of a specific PA intervention, as well as (2) clinical trials, cross-sectional studies, systematic reviews and meta-analyses.

### Screening & data extraction

All pertinent studies detected after searching the electronic databases were imported to a reference manager software (Citavi 6) and duplicates were removed. The study selection process was divided into three phases. Two independent reviewers (LC & JKR) screened the titles of the articles, followed by the abstracts, and finally the full-texts based on the inclusion criteria. All studies meeting the eligibility criteria were included in this review. Disagreement was resolved by consensus or by consulting a third author (CN). The following information was extracted by one reviewer (LC): first author’s name, publication year, study design, study setting, sample size, follow-up time, participant characteristics (e.g. age, sex, BMI) at baseline, assessment and type of PA (e.g. type, duration, intensity), assessment and type of outcomes of interest (i.e. obesity, CHD, diabetes and hypertension), and main results/ findings of the study (e.g. hazard ratios, relative risk). Extracted data were verified by another author (JKR).

### Quality assessment and risk of bias

The quality of included studies was evaluated independently by two authors (LC & JKR) using the 22-item Strengthening the Reporting of Observational Studies in Epidemiology (STROBE) statement version 4 [23]. Similarly, potential risk of bias of each study included in this systematic review was assessed through the Tool to Assess Risk of Bias in Cohort Studies [24] by the same authors (LC & JKR). Any discrepancies between the two reviewers were resolved by discussion or by consulting a third reviewer (CN).

## Results

**Fig. 1:**
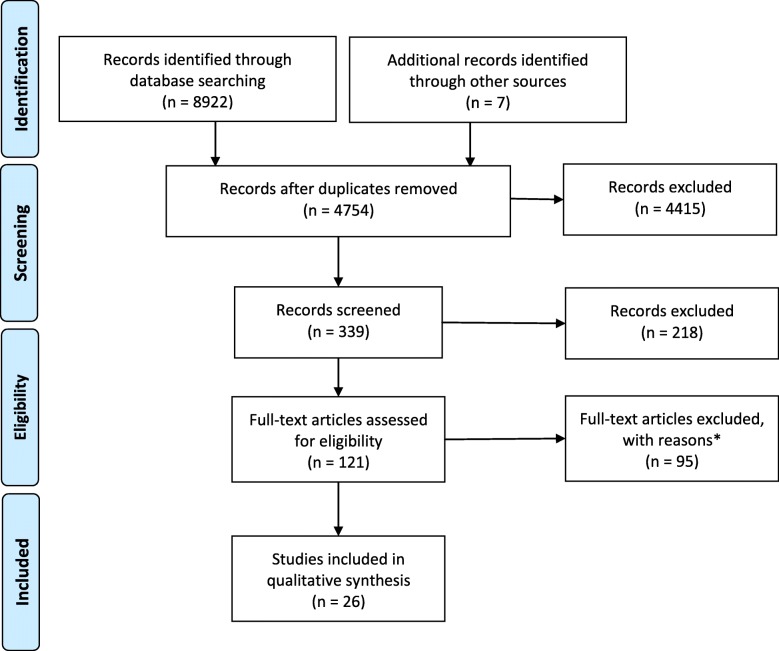
Flow Chart (modified based on [22]). * = main reasons for exclusion of studies (*n* = 35 other outcomes of interest; *n* = 24 other definition of PA; *n* = 10 other study designs; *n* = 6 follow-up time < 5 years; *n* = 20 other reasons)

Overall, we identified 8929 articles, of which 8903 articles were excluded as they did not meet the inclusion criteria as described above. The reader is referred to Fig. 1 for a flow chart summarizing the search process and number of studies at each step. Twenty-six studies were included in this review with a combined N of 1,145,298 participants, and follow-up times ranging between 5 and 34 years. Three articles examined the association between PA and incident obesity [25–27], eight studies examined the association between PA and incident CHD [28–35], nine studies examined the association between PA and incident diabetes [36–44], and four studies examined the association between PA and incident hypertension [45–48]. In addition, one study reported the association between both PA and diabetes as well as PA and hypertension [49], and another study reported the association between PA and CHD, diabetes, and hypertension [50].

### Association between PA and obesity

The studies included in this review showed an overall association between higher PA and lower risk of incident obesity. Two out of three studies reported a reduced risk of becoming obese for individuals with high PA levels as compared to low PA [25, 27]. One study showed an elevated risk (142%) of becoming obese for persons who were physically inactive [26]. The characteristics of the included studies are summarized in Table 1.

**Table 1:** Overview of longitudinal studies on the association between PA and the outcome of obesity (BMI ≥ 30 kg/m^2^)

Author	Country	Characteristics	Follow-up time	Predictor variable: Physical activity	Outcome of interest	Main results
Bell et al. (2014) [25]	UK, Whitehall II study	*N* = 3670, 73% male, 55.5 ± 6.0 years	10 years; Baseline 1997–1999 Follow-up: 2002–2004, 2007–2009	Self-reported, duration of MVPA (h/wk)	Incident obesity	OR [95% CI]: low level PA as reference^a^:
				­-Low: 0–1.5		-High level PA 0.64 [0.44, 0.93] after 5 years
				-Intermediate: 1.56–4.25		
				-High: 4.27–20.56		-High level PA 0.63 [0.45, 0.88] after 10 years
Montgomerie et al. (2014) [26]	Australia	*N* = 1521, 50.6% male, age 44.6 ± 16.22 years	2898.9 ± 402.29 days Baseline: 1999–2003, follow-up: 2004–2006, 2008–2010	Self-reported, score: frequency x time per session x intensity	Incident obesity	RR [95% CI]: Association between physical inactivity & incident obesity^b^:
				-Inactive: < 100 sedentary, 100–1600 low		
						-1.42 [1.03, 1.95] *p* = 0.030
				-Active: 1600–3200 moderate, > 3200 high		
Pavey et al. (2016) [27]	Australia	*N* = 2735 women, 24.6 (20.6–28.5) years	12 years Baseline: 2000 Follow-up: 2012	Self-reported, score (MET-min/wk)	Change in BMI category	OR [95% CI]: increasing cumulative PA with very low activity as reference^c^:
				-Very low (< 250)		
						-Transition to obesity: 0.73 [0.59, 0.90], *p* < .05 OR [95% CI], very high cumulative PA with very low activity as reference^c^:
				-Low (250 to < 500)		
				-Active (500 to < 1000)		
				-Very active (> 1000)		
						-Transition to obesity 0.52 [0.30, 0.92], p < .05

Abbreviation: *BMI* Body Mass Index, *CI* confidence interval, *h* hour, *MET* metabolic equivalent, *min* minutes, *MVPA* moderate-to-vigorous physical activity, *N* number of participants, *OR* odds ratio, *p p*-value, *PA* physical activity, *RR* relative risk, *SD* Standard deviation, *wk* week

^a^ Model adjusted for age, sex, ethnicity; ^b^ Model adjusted for age, sex, chronic conditions (diabetes, asthma, chronic obstructive pulmonary disease, cardiovascular disease and mental health); ^c^ Model adjusted for educational level, area of residence, number of children, occupation, work time walking, work time in heavy labor, smoking status, alcohol consumption, energy intake, dieting, oral contraceptive pill use, number of chronic conditions

### Association between PA and CHD

Overall, there was an association between higher levels or amount of PA and a decreased risk of incident CHD. Seven out of nine studies reported a reduced risk of new onset of CHD with increasing PA levels as compared to low or no PA [28, 32–35], whereas one study revealed an association between PA and decreased CHD risk only for vigorous intensity PA [29]. One study did not find a significant association for occupational PA and CHD risk [30]. Two out of nine studies examined the impact of change in PA levels over time as predictor variable and failed to detect a significant association with incident CHD [31, 33]. Please refer to Table 2 for a summary of studies on PA and incident CHD.

**Table 2:** Overview of longitudinal studies on the association between PA and the outcome of CHD

Author	Country	Characteristics	Follow-up time	Predictor variable: Physical activity	Outcome of interest	Main results
Chomistek et al. (2016) [28]	USA, Nurses’ Health Study II (NHSII)	*N* = 97,230 women, 36.6 ± 4.6 years	20 years Baseline: 1991	Self-reported LTPA (MET-h/wk, in quintiles) - < 1	Incident CHD (nonfatal MI, fatal CHD)	HR [95% CI] of CHD event for total LTPA^a^:
						- < 1: 1.0 (reference)
						- 1–5.9: 0.86 [0.68, 1.08]
			Follow-up: 2011	- 1–5.9		- 6–14.9: 0.66 [0.52, 0.84]
				- 6–14.9		
				- 15–29.9		- 15–29.9: 0.48 [0.36, 0.63]
				- ≥30		- ≥30: 0.53 [0.41, 0.70]
						Similarly, increasing MET-h/wk were associated with a decreased risk of incident CHD when looking at moderate-intensity PA only, as well as looking at vigorous-intensity PA only.
Delaney et al. (2013) [29]	USA, Multi-Ethnic Study of Atherosclerosis (MESA)	*N* = 5656, 47.4% male, 61.3 ± 9.9 years, BMI 28.3 ± 5.4 kg/m^2^	5 years Baseline 2000–2002	Self-reported PA (total min/d, total MET-min/d)	Incident CAC	RR [95% CI] of PA and incident CAC^b^:
						- Vigorous activity: 0.97 [0.94, 1.00], *p* = 0.048
			Follow-up: 2005–2007			No association between intentional, sedentary, MVPA and conditioning PA and incident CAC.
Ferrario et al. (2018) [30]	Italy, MONICA, PAMELA, SEMM	*N* = 3574 men, 25–64 years	Median 14 years (IQR 12.9–15.9)	Self-reported, OPA score 1–5 (tertiles, cut-offs at 2.5, 3.125), SpPA index (min/wk of MPA or VPA based on METs of task)	Incident CHD (first acute coronary event as MI, acute coronary syndrome or coronary revascularization)	HR [95% CI] of first CHD event (fatal or non-fatal) by OPA group^c^:
						- Low: 1.66 [1.06, 2.59]
			Baseline: 1989–1996			- Intermediate: 1.0 (reference)
						- High: 1.18 [0.72, 1.94]
			Follow-up: 2008	- Poor: 0		HR [95% CI] of first CHD event (fatal or non-fatal) by SpPA group^c^:
				- Intermediate: 1–149 MPA or 1–74 VPA or 1–149 MPA plus VPA		
						- Poor: 1.0 (reference)
						- Intermediate: 0.81 [0.50, 1.32]
				- Recommended: ≥150 MPA or ≥ 75 VPA or ≥ 150 MPA plus VPA		- Recommended: 0.58 [0.30, 1.12]
Jefferis et al. (2014) [31]	UK, British Regional Heart Study	*N* = 3320 men, 68.3 ± 5.4 years	Median 11 years Baseline 1998–2000 Follow-up: 2010	Self-reported usual PA (score)	Incident CHD (First fatal or nonfatal MI events, ICD-9 Code 410–414, ICD − 10 Code I21-I23, I252)	HR [95% CI] of first CHD event (fatal or non-fatal) by PA group^d^:
				- Inactive (0–2)		
				- Occasional (3–5)		- None: 1.0 (reference)
				- Light (6–8)		- Occasional: 0.52 [0.34, 0.79]
				- Moderate (9–12)		- Light: 0.47 [0.30, 0.74]
				- Moderately vigorous (13–20)		- Moderate: 0.51 [0.32, 0.82]
						- Moderately vigorous and vigorous: 0.44 [0.29, 0.65]
				- Vigorous (> 21)		
				Change in PA (1996–2000)		*p* = 0.004
				- Always inactive		HR [95% CI] of first CHD event (fatal or non-fatal) by change in PA group^d^:
				- Became inactive		
				- Became active		- Always inactive: 1.0 (reference)
				- Always active		- Became inactive: 0.87 [0.53, 1.45]
						- Became active: 0.86 [0.55, 1.35]
						- Always active: 0.73 [0.53, 1.02]
Koolhaas et al. (2016) [32]	Netherlands, The Rotterdam Study	*N* = 5901, median age 67 years	15 years Baseline 1997–2001 Follow-up: 2012	Self-reported PA, tertiles (median (range) MET-h/wk in total PA)	Incident CHD (fatal or nonfatal MI, surgical/ percutaneous coronary revascularization procedure)	Total PA and risk of incident CHD event^e^: HR [95% CI]
						- Tertile 1: 1.0 (reference)
				- Tertiles 1: 42.0 (≤61.4) ≙ 1.5 h/d at 4 METs		- Tertile 2: 0.76 [0.63, 0.92]
						- Tertile 3:0.69 [0.57, 0.84]
				- Tertiles 2: 77.5 (61.5–96.9) ≙ 2.8 h/d at 4 METs		Per 10 MET-h/wk: 0.96 [0.94, 0.98]. p overall = < 0.001.
				- Tertiles 3: 126.7 (≥97.0) ≙ 4.5 h/d at 4 METs		
Petersen et al. (2012) [33]	Denmark, Copenhagen City Heart Study	*N* = 10,443, 57% female, median age 58 years	Baseline 1976–78	Self-reported LTPA	Incident CHD fatal and non-fatal cases (MI: ICD-8 Code 410, ICD-10 Code I21–22; IHD: ICD-8 Code 410–414, ICD-10 Code I20–25)	HR [95% CI] of IHD by PA change^f^: Women | Men
				- Sedentary		
			Follow-up: 1981–1983, 2008	- Light		- -2: 1.60 [1.02, 2.32] | 1.33 [0.97, 1.83]
				-Moderate		
				- Vigorous		- -1: 1.28 [1.10, 1.49] | 1.12 [0.96, 1.31]
				Change in PA categories (1976/78–1981/83)		
						- 0: 1.0 (reference)
				- -2/−3 categories		- 1: 0.97 [0.85, 1.12] | 1.09 [0.96, 1.25]
				- -1 category		
				- 0 (stable)		- 2: 1.01 [0.75, 1.38] | 1.16 [0.89, 1.51]
				- + 1 category		
				- + 2/+ 3 categories		HR [95% CI] of MI by PA change^f^: Women | Men
						- -2: 1.56 [0.89, 2.75] | 1.74 [1.17, 2.60]
						- -1: 1.30 [1.03, 1.65] | 1.13 [0.91, 1.39]
						- 0: 1 (reference)
						- 1: 0.98 [0.79, 1.22] | 1.14 [0.95, 1.36]
						- 2: 1.08 [0.67, 1.75] | 1.30 [0.92, 1.84]
Soares-Miranda et al. (2016) [34]	USA	*N* = 4207, 39% males, 72.5 ± 5.5 years	10 years Baseline: 1989	Self-reported LTPA (kcal/wk), exercise intensity	Incident CHD (fatal & nonfatal MI & CHD death)	HR [95% CI] for exercise intensity^g^, none as reference:
			Follow-up: 1999	- None		- Low: 0.56 [0.43, 0.72], *p* < 0.001
				- Low		- Moderate: 0.53 [0.41, 0.69], p < 0.001
				- Moderate		- High: 0.47 [0.32, 0.69], p < 0.001
				- High		
Tikkanen et al. (2018) [35]	UK	*N* = 502,635, 54% female, 56.5 ± 8.1 years	Median 6.1 years Baseline: 2006–10	Self-reported (MET-h/wk)	Incident CHD (ICD-9 Code 410–411, ICD-10 Code I20.0, I21, I22)	HR [95% CI] association CHD and PA level^h^: - 0.95 [0.93, 0.97], *p* < 0.001
			Follow-up: 2015–2016			
Williams & Thompson (2012) [50]	USA	*N* = 47,921	Median 6.2 years	Self-reported PA MET-h/d	Incident CHD (MI, CABG, percutaneous coronary intervention, and angina pectoris)	Greater MET-h/d is associated with lower risk of incident CHD: HR [95% CI]^i^
				- Light		
			Baseline: 1998–1999	- Moderate		- Running: 0.955 [0.91, 1.00]
				- Vigorous		- Walking: 0.907 [0.839, 0.98]
			Follow-up: 2006			- Other vigorous: 0.99 [0.966, 1.02]
						- Other moderate: 0.98 [0.927, 1.04]
						- Other light: 0.98 [0.807, 1.197]

Abbreviation: *BMI* Body Mass Index, *CABG* coronary artery bypass graphs, *CAC* coronary artery calcification, *CHD* coronary heart disease, *CI* confidence interval, *d* day, *h* hour, *HDL* high density lipoprotein, *HR* hazard ratio, *ICD* International Classification of Diseases, *IHD* ischemic heart disease, *IQR* interquartile range, *kcal* kilocalories, *LTPA* leisure time physical activity, *MET* metabolic equivalent, *MI* Myocardial Infarction, *min* minutes, *MPA* moderate physical activity, *MVPA* moderate to vigorous physical intensity, *N* number of participants, *OPA* occupational physical activity, *p* p-value, *PA* physical activity, *RR* relative risk, *SpPA* sport physical activity, *VPA* vigorous physical activity, *wk* week

^a^ Model adjusted for age; ^b^ Model adjusted for age, sex, ethnicity, BMI, pack years of smoking, family history of MI, hypertension, dyslipidemia, diabetes, education, alcohol use, current smoking status, education, income, health insurance status; ^c^: Model adjusted for age, cohort, educational level, OPA and SpPA; ^d^Model adjusted for age and region; ^e^ Model adjusted for age and sex; ^f^ Model adjusted for physical activity level in 1976–1978, age, education, smoking habits, alcohol consumption, BMI, diabetes, cholesterol, blood pressure lowering therapy in 1981–1983; ^g^ Model adjusted for age, sex, race, education, income, clinical sites, smoking, BMI; ^h^ Model adjusted for age, sex, region; ^i^ Model adjusted for baseline age (age, age2), sex, race, education, smoking, intakes of red meat, fruit, alcohol

### Association between PA and diabetes

The studies included in this review provide evidence of an association between increasing PA levels and a decreased risk of incident diabetes. Nine out of 11 studies reported a gradual inverse association between increasing PA levels with up to high/vigorous-intensity and a decreased risk of incident diabetes [36–39, 40–42, 44, 50], whereas one study revealed an association only between moderate intensity PA and reduced diabetes risk [39]. Two out of 11 studies reported an increased risk of incident diabetes (179 and 145%, respectively) for participants engaging in low amount of leisure time PA as compared to the highly active reference group [43, 49]. For a summary of included studies on the association between PA and diabetes please refer to Table 3.

**Table 3:** Overview of longitudinal studies on the association between PA and the outcome of diabetes

Author	Country	Characteristics	Follow-up time	Predictor variable: Physical activity	Outcome of interest	Main results
Carlsson et al. (2013) [36]	Sweden (Swedish Twin Registry)	*N* = 23,539	Baseline: 1967–1972	Self-reported LTPA	Incident type 2 diabetes	Risk of type 2 diabetes decreased with PA: HR [95% CI]
				- Low		- Low: 1.0 (reference)
			Follow-up: 1998–2002	- Moderate		- Moderate: 0.77 [0.61, 0.96]
				- high		- High: 0.53 [0.37, 0.75]
Elwood et al. (2013) [40]	UK (Caerphilly Prospective Study CaPS)	*N* = 2235 men, 45–59 years	30 years	Self-reported PA	Incident diabetes (self-reported)	OR [95% CI] for regular activity and incident diabetes
			Baseline: 1979–1983			- 0.63 [0.46, 0.85]
			Follow-up: 1984–1988, 1989–1993, 1993–1997, 2009			
Grøntved et al. (2014) [41]	USA (Nurses’Health Study NHS I and II)	*N* = 99,316 women	8 years	Self-reported PA (time spent on resistance exercise per week, lower intensity muscular conditioning exercises (yoga, stretching, toning), aerobic MVPA)	Incident diabetes (self-report confirmed using standardized criteria; validated in sub-sample through medical chart review)	RR [95% CI] for incident diabetes for aerobic MVPA
			Baseline (NHS I): 2000			- None: 1.0 (reference)
						- 1–29 min: 0.83 [0.74, 0.92]
			Baseline (NHS II): 2001			- 30–59 min: 0.73 [0.65, 0.82]
						- 60–150 min: 0.66 [0.60, 0.73]
			Follow-up (NHS I): 2008	- None		- ≥ 150 min: 0.46 [0.41, 0.50]
				- 1–29 min/wk		- Trend: p < 0.001
			Follow-up (NHS II): 2009	- 30–59 min/wk		Engaging in at least 150 min/wk of aerobic MVPA and at least 60 min/wk of muscle-strengthening activities was significantly associated with lower risk of incident diabetes compared with being inactive (pooled RR = 0.33 [0.29, 0.38]).
				- 60–50 min/wk		
				- > 150 min/wk		
Hjerkind et al. (2017) [37]	Norway (Nord- Trøndelag Health Study)	*N* = 38,413 with information on PA, 47% males	11 years	Self-reported LTPA	Incident diabetes (self-reported; validated through medical record)	Risk of diabetes decreased with PA^a^: RR [95% CI] Women | Men:
			Baseline: 1984–1986	- Low		
				- Medium		- Low: 1.0 (reference)
			Follow-up: 1995–1997	- High		- Medium: 0.81 [0.65, 1.00] | 0.80 [0.66, 0.98]
						- High: 0.76 [0.61, 0.95] | 0.65 [0.51, 0.84] *p* = 0.01 | *p* < 0.01
						Gradual inverse association between frequency, duration, intensity and risk of incident diabetes for males
						Gradual inverse association between frequency, intensity and risk of incident diabetes for females
Ekelund et al. (2012) [42]	8 European countries (EPIC–InterAct Study)	*N* = 11,669 men, 15,695 women	Median 12.3 years	Self-reported PA (OPA, LTPA)	Incident diabetes	A one level difference in PA (e.g. between inactive and moderately inactive) was associated with a 13% relative reduction in risk of incident diabetes in males (HR [95% CI] 0.87 [0.80, 0.94]) and 7% risk reduction in females (0.93 [0.89, 0.98])^b^
				- Inactive		
		*N* = 15,934 subcohort (6009 men, 9925 women)	Baseline: 1991	- Moderately inactive		
			Follow-up: 2007	- Moderately active		
				- Active		
						Increased risk of incident diabetes associated with lower levels of PA evident across BMI strata in both sexes, with the exception of obese women
Jefferis et al. (2012) [38]	UK	*N* = 3012 men, 68.3 years	Median 7.1 years	Self-reported PA	Incident type 2 diabetes (self-report included after validation through medical record)	Risk of diabetes decreased with PA: Dose-response association^c^: HR [95% CI]
				- None		
			Baseline: 1996, 1998–2000 Follow-up:	- Occasional		- None: 1.0 (reference)
				- Light		- Occasional: 0.54 [0.31, 0.96]
			2006	- Moderate		- Light: 0.34 [0.18, 0.65]
				- Moderately vigorous		- Moderate: 0.33 [0.17, 0.65]
				- Vigorous		- moderately vigorous: 0.32 [0.16, 0.61]
						- vigorous: 0.26 [0.13, 0.53] p < 0.01
						Taking up at least moderate intensity PA also associated with lower risk of diabetes.
Koloverou et al. (2017) [39]	Greece (Attica Study)	*N* = 1485, 49% males	10 years	Self-reported PA (MET-min/wk)	Incident diabetes (measured in biological sample or self-reported)	Moderate intensity PA associated with lower risk of incident diabetes^d^: OR [95% CI]
			Baseline: 2001–2002	- Very low ≤150		
				- Low = 150–330		- Very low: 1.0 (reference)
			Follow-up: 2011–2012	- Moderate = 331–1484		- Low: 0.77 [0.41, 1.49]
				- High ≥1484		- Moderate: 0.47 [0.24, 0.93]
						- High: 1.04 [0.59, 1.82]
Medina et al. (2018) [49]	Mexico (Mexico City Diabetes Study)	*N* = 1883, median 45 years (IQR 39–52); 42.7% males	Median 14.4 person years	Self-reported PA (occupational, leisure, total PA in MET-min/wk of MVPA)	Incident type 2 diabetes (measured, self-reported, taking medication)	Participants with leisure PA < 1 MET-min/wk had increased risk of incident diabetes (HR 1.45 [95% CI: 1.10, 1.92]) as compared to reference group (≥ 1200 MET-min/wk of MVPA; *p* = 0.008)^e^
			Baseline: 1989–1990			
				- 1 = < 1		
						No association between occupational and total PA and diabetes risk.
			Follow-up: 1993–1994, 1997–1998, 2008–2009	- 2 = 1–599.9		
				- 3 = 600–1199.9		
				- 4 = ≥ 1200		
Mehlig et al. (2014) [43]	Sweden	*N* = 1448 women, 38–60 years	34 years	Self-reported LTPA	Incident diabetes	LTPA is associated with an elevated risk in incident diabetes: HR [95% CI]^f^
			Baseline: 1968–1969	- Almost inactive: low LTPA		
				- Some PA at least 4 h/wk		- Non-obese, active: 1.0 (reference)
			Follow-up: 1974–1975, 1980–1981, 1992–1993, 2000–2001, 2000	- Regular exercise		- Non-obese, inactive: 1.79 [1.15, 2.79]
				- Regular training and competitive sports		- Obese, active: 2.43 [1.44, 4.09]
						- Obese, inactive: 11.7 [6.28, 21.8]
Shi et al. (2013) [44]	China	*N* = 51,464 men, 54.1 ± 9.3 years	Median 5.4 years	Self-reported PA MET level (in quintiles)	Incident diabetes (self-reported)	Total PA is associated with a reduced risk in incident diabetes: HR [95% CI] for MET level^g^
			Baseline: 2002–2006	- Q1 < 4.3		- Q1: 1.0 (reference)
				- Q2 4.3–6.5		- Q2: 0.84 [0.72, 0.99]
			Follow-up: 2004–2008, 2008–2011	- Q3 6.5–8.9		- Q3: 0.72 [0.61, 0.85]
				- Q4 8.9–12.1		- Q4: 0.66 [0.55, 0.78]
				- Q5 ≥ 12.1		- Q5: 0.65 [0.54, 0.77]
Williams & Thompson (2012) [50]	USA	*N* = 48,116	Median 6.2 years	Self-reported PA MET-h/d	Incident diabetes	Greater MET-h/d is associated with lower risk of incident diabetes: HR [95% CI]^h^
				- Light		
			Baseline: 1998–1999	- Moderate		- Running: 0.879 [0.83, 0.929]
				- Vigorous		- Walking: 0.877 [0.82, 0.93]
			Follow-up: 2006			- Other vigorous: 0.98 [0.95, 1.007]
						- Other moderate: 0.969 [0.908, 1.02]
						- Other light: 0.99 [0.736, 1.12]

Abbreviation: BP - *CI* confidence interval, *d* day, *h* hour, *HR* hazard ratio, *IQR* interquartile range, *LTPA* leisure time physical activity, *MET* metabolic equivalent, *min* minutes, *MVPA* moderate to vigorous physical activity, *N* number of participants, *OPA* occupational physical activity, *OR* odds ratio, *PA* physical activity, *RR* relative risk, *wk* week

^a^: Model adjusted for age; education, alcohol frequency in the past 2 weeks, smoking, blood pressure medication use, prevalent cardiovascular disease, BMI, PA summary score; b Model adjusted for study center, education, smoking status, alcohol consumption, energy intake, BMI; ^c^ Model adjusted for age & region; ^d^ Model adjusted for age, sex, family history of diabetes, hypertension, hypercholesterolemia, smoking status, education, physical activity, waist circumference, adherence to the Mediterranean diet, fasting glucose, triglycerides; ^e^ Model adjusted for sex, age, education levels, marital status, current smoking, alcohol intake, total energy intake, parent history of diabetes, sleeping hours, leisure/working MET-min/wk; ^f^ Model adjusted for baseline covariates age, education, smoking, consumption of alcohol, triglycerides, hypertension, parental history of diabetes (diabetes only); ^g^ Model adjusted for age at interview, energy intake, smoking, alcohol consumption, education level, occupation, income level, hypertension, family history of diabetes; ^h^ Model adjusted for baseline age (age, age2), sex, race, education, smoking, intakes of red meat, fruit, alcohol, preexisting CHD at baseline

### Association between PA and hypertension

Overall, there was no consistent association between PA and incident hypertension. Three out of six studies reported a gradual inverse association between PA levels (running and walking, moderate and moderate-vigorous PA) and incident hypertension [46, 48, 50], whereas one study found an association only for a specific age group (51–60 years) [48]. Two out of six studies found no significant association between PA and incident hypertension [45, 47]. One out of six studies reported an increased risk of incident hypertension (137%) for persons with low leisure time PA as compared to the highly active reference group [49]. Please refer to Table 4 for an overview of included studies.

**Table 4:** Overview of longitudinal studies on the association between PA and the outcome of hypertension

Author	Country	Characteristics	Follow-up time	Predictor variable: Physical activity	Outcome of interest	Main results
Cohen et al. (2012) [48]	USA (Nurses’ Health Study I cohort)	*N* = 78,590 women; 49 years (IQR 44–56)	20 years	Self-reported PA	Incident hypertension (self-reported; validated in NHS I cohort)	Association between PA and incident hypertension varies by age (p-value for interaction < 0.001).
			Baseline: 1984	METs/wk for vigorous exercise in quintiles (Q1–5)		
			Follow-up: 2004			HR [95% CI] lowest for PA Q5 as compared to Q1.
						- Age ≥ 50: Q1 1.0 (reference); Q2 1.00 [0.91, 1.11]; Q3 1.03 [0.94, 1.14]; Q4 1.01 [0.91, 1.12]; Q5 0.87 [0.78, 0.97]
						- Age 51–60: Q1 1.0 (reference); Q2 0.94 [0.88, 1.00]; Q3 0.94 [0.88, 1.00]; Q4 0.91 [0.85, 0.97]; Q5 0.86 [0.80, 0.92]
						- Age ≥ 61: Q1 1.0 (reference); Q2 1.03 [0.97, 1.09]; Q3 0.98 [0.93, 1.04]; Q4 0.99 [0.93, 1.05]; Q5 0.95 [0.90, 1.01]
Lu et al. (2015) [45]	China	*N* = 1009, 35.48 ± 0.19 years, 63.4% males	Median 4.7 years	Self-reported PA	Incident hypertension	No significant association between PA and risk of hypertension^a^: HR [95% CI]
				- Frequent		
			Base line: 2004	- Occasional		- Occasional: 0.74 [0.40, 1.39]
			Follow-up: 2012	- Everyday		- Frequent: 0.96 [0.51, 1.83]
						- Everyday: 1.0 (reference)
Medina et al. (2018) [49]	Mexico (Mexico City Diabetes Study)	*N* = 1541, median 45 (IQR 39–52) years; 45.1% males	Median 11.8 years	Self-reported PA (occupational, leisure, total activity in MET-min/wk of MVPA)	Incident hypertension (measured by study team)	Participants with < 1 MET-min/wk of leisure (HR 1.37 [95% CI 1.07, 1.75], *p* = 0.015) or occupational MVPA (HR 1.52 [1.17, 1.97], *p* = 0.001) had increased risk of hypertension as compared to reference group (≥ 1200 MET-min/wk)^b^
			Baseline: 1989–1990			
				- 1 = < 1		
			Follow-up: 1993–1994, 1997–1998, 2008–2009	- 2 = 1–599.9		No association was observed between total PA and hypertension.
				- 3 = 600–1199.9		
				- 4 = ≥ 1200		
Pavey et al. (2013) [46]	Australia (Australian Longitudinal Study on Women’s Health)	*N* = 11,285 women, mean age 49.5 years	Baseline: 1998	Self-reported PA (MET-min/wk)	Occurrence of hypertension (self-reported)	OR [95% CI] for hypertension declined with increasing PA volume; decline slightly greater in MVPA than MPA group MPA^c^ | MVPA^c^:
			Follow-up: 2001, 2004, 2007, 2010	- None		
				- > 0- < 250		
				- 250- < 500		- None: 1.0 (reference)
				- 500- < 1000		- > 0- < 250: 0.92 [0.83, 1.02] | 0.87 [0.63, 1.04]
				- 1000- < 1500		- 250- < 500: 0.90 [0.81, 1.00] | N.A.
				- 1500- < 2000		- 500- < 1000: 0.82 [0.75, 0.91] | 0.73 [0.62, 0.86]
				- > 2000		- 1000- < 1500: 0.74 [0.66, 0.82] | 0.65 [0.55, 0.76]
				- Inactive		- 1500- < 2000: 0.78 [0.68, 0.90] | 0.63 [0.54, 0.74]
				- Moderate (MPA)		- > 2000: 0.80 [0.70, 0.93] | 0.56 [0.49, 0.64]
				- Moderate and vigorous activity (MVPA)		
Stenehjem et al. (2018) [47]	Norway (Nord- Trøndelag Health Study)	*N* = 21,892, 42.7% males	11 years	Self-reported LTPA	Incident hypertension (measured by study team)	Risk of hypertension not associated with LTPA total score^d^: RR [95% CI]
			Baseline: 1984–1986	Total score		
				- Low		Women | Men
			Follow-up: 1995–1997	- Medium		- Low: 1.0 (reference)
				High		- Medium: 0.98 [0.92, 1.05] | 0.96 [0.90, 1.03]
				Frequency (per wk)		- High: 0.96 [0.90, 1.01] | 0.97 [0.90, 1.03] *p* = 0.138 | *p* = 0.276
				- None		
				- < 1		Frequency of PA associated with reduced risk of hypertension only in males (≥4/wk: RR 0.87 [0.78, 0.98]). Obese males with high PA have lower risk of hypertension (RR 1.16 [0.79, 1.70]) than obese males with low PA (RR 1.50 [1,27, 1.77]).
				- 1		
				- 2–3		
				- ≥4		
				Intensity		Obese females with low PA have increased risk of hypertension (RR 1.55 [1.35, 1.77]).
				- None		
				- Low		
				- Medium/high		
Williams & Thompson (2012) [50]	USA	*N* = 43,893	Median 6.2 years	Self-reported PA	Incident hypertension	Greater MET-h/d is associated with lower risk of incident hypertension: HR [95% CI]^e^
				MET-h/d		
			Baseline: 1998–1999	- Light		- Running: 0.958 [0.94, 0.97]
				- Moderate		- Walking: 0.928 [0.899, 0.957]
			Follow-up: 2006	- Vigorous		- Other vigorous: 0.98 [0.97, 0.99]
						- Other moderate: 0.997 [0.976, 1.018]
						- Other light: 0.886 [0.739, 1.006]

Abbreviation: *CI* confidence interval, *d* day, *h* hour, *HR* hazard ration, *IQR* interquartile range, *LTPA* leisure time physical activity, *MET* metabolic equivalent, *min* minutes, *MPA* moderate physical activity, *MVPA* moderate to vigorous physical activity, *N* number of participants, *N.A.* not available, *p* p-value, *PA* physical activity, *RR* relative risk, *SE* standard error, *wk* week

^a^: Model adjusted for age, gender and follow-up time; ^b^ Model adjusted for sex*time, age, education levels, marital status, current smoking, alcohol intake*time, total energy intake, sleeping hours, leisure/ working METs/min/wk; ^c^ Model adjusted for sociodemographic (age, education, marital status, area of residence), behavioral (smoking, alcohol, and sitting), chronic conditions covariates; ^d^ Model adjusted for age, marital status, education, smoking, alcohol frequency last 2 weeks, BMI, PA summary score; ^e^ Model adjusted for baseline age (age, age2), sex, race, education, smoking, intakes of red meat, fruit, alcohol, preexisting CHD at baseline

## Discussion

The purpose of this research was to review studies published after January of 2012 and up to February of 2019 that investigated the long-term association between PA and new onset of obesity, CHD, diabetes and hypertension. Overall, we observed an association between PA and a decreased risk of incident obesity, CHD and diabetes but not hypertension. This is in line with systematic reviews published by our group and others that also found beneficial associations of PA with overweight/obesity, CHD and diabetes [20, 51–53]. Furthermore, it is also consistent with our hypothesis derived from both interventional and observational studies [54–57].

The included studies that examined the association between PA and incident hypertension reported conflicting results. This is partly in accordance with two other meta-analyses [58, 59]. One meta-analysis reported a reduction of incident hypertension by 6% for each 10 metabolic equivalent of task hours per week increment of leisure time PA [58]. However, another meta-analysis detected an inverse association for recreational PA and incident hypertension but not for occupational PA [59]. Of note, the causes of hypertension are multifactorial and the way they interact and ultimately contribute to the development of hypertension is unclear. Thus, potential mechanisms for prevention of hypertension through PA also remain unclear.

Some studies included in this review also reported findings stratified by sex and body weight. For example, one study observed a gradual inverse association between frequency, duration and intensity of PA and risk of incident diabetes in males, but only a gradual inverse association between frequency and intensity of PA and risk of incident diabetes in females [37]. Additionally, another study reported that, while overall PA irrespective of body weight was not associated with the outcome of incident hypertension, obese males with high PA had a significantly lower risk of hypertension than obese males with low PA [47].

The quality of included studies was independently assessed by two reviewers and was rated as moderate to good, with scores ranging between 16 and 22 (total range: 0–22). This is not surprising since we only included studies published in or after 2012 that may already have followed quality guidelines on reporting findings of observational studies such as STROBE [23]. The potential risk of bias was rated moderate to poor and there were several concerns that warrant brief discussion: 1) All studies included in this review assessed PA through self-reported questionnaires which may be prone to recall bias. However, given the large sample sizes and since the baseline measurements of PA of many studies took place several years or even decades ago, objective measurement of PA might not have been feasible. There is good reason to believe that more longitudinal studies using novel objective techniques such as accelerometry (e.g. [60, 61]) will become available in the near future. 2) The studies differed regarding the assessment of the outcomes of interest, e.g. some studies objectively measured blood glucose levels or blood pressure (e.g. [47, 49]) whereas others relied on self-reported information by the study participants and/or medical chart review (e.g. [40, 48]). 3) The studies differed in terms of adjustment for potential confounders and mediators which makes a comparison of findings between studies difficult. 4) Five studies were conducted only among males [30, 31, 38, 40, 44] and five studies were conducted only among females [28, 41, 43, 46, 48].

We did not investigate potential mechanisms underlying the associations between PA and incident obesity, diabetes and CHD. However, it has previously been postulated that there are acute and chronic effects of PA on insulin resistance, which may account for improvements in insulin action and decreased blood glucose levels as a response to engagement in PA [62]. Additionally, PA impacts energy balance by increasing total energy expenditure, which in turn causes an energy deficit and may lead to lower body weight [63]. Stimulating responses in adipose and body tissues by PA may also influence total energy balance and body composition [63]. Furthermore, studies suggest that regular PA increases capillarization and may reverse endothelial alterations, which is a major risk factor for CHD [64].

The strengths of this review are the rigorous search and selection strategy following published guidelines and conducted by two reviewers. Also, both quality and potential risk of bias were assessed by two authors independently. We deliberately focused on studies published after January of 2012 in order to provide an update of a systematic review previously published by our group [20]. In addition, even though unintentional, our review included studies from various countries such as Sweden, Norway, UK, Greece, Mexico, China, Australia, Italy, US, the Netherlands, Germany, France, Spain and Denmark which may add to the generalizability of our observations. The major limitations of this review pertain to the relatively small number of included studies. This may be due to the fact that we only included studies with large sample size (*N* ≥ 500) and relatively long follow-up time of ≥5 years. However, we believe that these criteria ensure validity of our conclusions and a higher probability of generalizability of the study findings. In addition, a large body of research on PA and overweight/obesity published after 2012 focused on change in BMI or body weight over time. We opted to not include these studies in our review as we chose our outcome of interest to be incident obesity, and information on change in BMI or body weight over time is thus not sufficient. For instance, a person could be underweight at baseline and an increase in BMI or weight might actually reflect progression to a healthier body constitution. At the same time, we also acknowledge that particularly obesity and hypertension are conditions for which individuals can take action to improve, i.e., a person develops incident obesity but may be able to decrease body weight in order to progress back to overweight or normal weight. Furthermore, our search terms may have been too narrow or not comprehensive enough and there may be published studies that we were not able to identify. However, in order to compensate for this potential shortcoming, we also manually searched bibliographies of included studies. Finally, the studies differed with regard to the depth of investigating PA variables. As such, PA was only one of many predictors in some studies (e.g. [36, 42]) and thus only one finding related to PA and the outcome of interest was reported. Whereas in other studies, the association between various PA parameters (e.g. type, intensity, frequency, duration) and the outcome of interest was examined (e.g. [30, 47]).

More research to untangle the association and potential underlying mechanisms between PA and the outcome of incident overweight, CHD, diabetes and hypertension is needed, preferably using prospective cohort studies with large sample sizes, long follow-up and objective measurement of both predictor variable (i.e. PA) and the outcomes of interest. In addition, meta-analytic approaches to address research questions pertaining the association between PA and various health outcomes are warranted.

## Conclusion

Overall, this systematic review replicates, updates, and extends the growing body of research on the associations between PA and incident obesity, CHD and diabetes. No clear association between PA and reduced risk for hypertension was detected. This review emphasises the contribution of PA in the prevention of various chronic diseases. Reducing the risk of new onset of NCDs and thereby reducing the economic burden on health systems is of high importance to societies worldwide. Regional and global action plans and preventive strategies (e.g. [65]) should highlight the beneficial impact of regular PA and support national governments in the implementation of concrete actions towards achieving a higher engagement in PA among individuals across all ages.

## Data Availability

All data generated or analyzed during this study are included in this published article.

## Abbreviations

BMI: Body mass index
CHD: Coronary heart disease
NCDs: Non-communicable diseases
PA: Physical activity
PRISMA: Preferred Reporting Items for Systematic Reviews and Meta-Analysis
